# Proximal gastrectomy with gastric tube reconstruction or jejunal interposition reconstruction in upper-third gastric cancer: which offers better short-term surgical outcomes?

**DOI:** 10.1186/s12893-021-01239-7

**Published:** 2021-07-05

**Authors:** Zhiguo Li, Yan Ma, Guiting Liu, Ming Fang, Yingwei Xue

**Affiliations:** 1 Department of Gastroenterological Surgery, Harbin Medical University Cancer Hospital, Nangang District, No.150 Haping Rd, Harbin, 150081 China; 2 Department of Thoracic Surgery, Mudanjiang Medical College, Hongqi Hospital, Mudanjiang, 157011 China; 3 Department of Oncology, Nenjiang People’s Hospital, Nenjiang, China

**Keywords:** Gastric cancer, Proximal gastrectomy, Jejunal interposition, Gastric tube, Surgical short-term outcomes

## Abstract

**Objective:**

Proximal gastrectomy acts as a function-preserving operation for upper-third gastric cancer. The aim of this study was to compare the short-term surgical outcomes between proximal gastrectomy with gastric tube reconstruction and proximal gastrectomy with jejunal interposition reconstruction in upper-third gastric cancer.

**Methods:**

A retrospective review of 301 patients who underwent proximal gastrectomy with jejunal interposition (JI) or gastric tube (GT) at Harbin Medical University Cancer Hospital between June 2007 and December 2016 was performed. The Gastrointestinal Symptom Rating Scale (GSRS) and Visick grade were used to evaluate postgastrectomy syndromes. Gastrointestinal fiberoscopy was used to evaluate the prevalence and severity of reflux esophagitis based on the Los Angeles (LA) classification system.

**Results:**

The JI group had a longer operation time than the GT group (220 ± 52 vs 182 ± 50 min), but no significant difference in blood loss was noted. Compared to the GT group, the Visick grade and GSRS score were significantly higher. Reflux esophagitis was significantly increased in the GT group compared with the JI group.

**Conclusion:**

Proximal gastrectomy is well tolerated with excellent short-term outcomes in patients with upper-third gastric cancer. Compared with GT construction, JI construction has clear functional advantages and may provide better quality of life for patients with upper-third gastric cancer.

## Introduction

Gastric cancer is one of most common types of solid tumors and the leading life-threatening cancer [1]. In 2018, more than one million new cases of gastric cancer were estimated to have occurred according to GLOBOCAN. Gastric cancer is the fifth most common malignancy of all cancers and the third leading cause of cancer mortality and all cancer deaths. Global Cancer Statistics 2018: GLOBOCAN estimates the worldwide incidence and mortality for 36 cancers in 185 countries [2]. In recent years, the incidence of upper-third gastric cancer (proximal gastric cancer), including esophagogastric junction cancer, has been increasing, and the number of cases of early-stage proximal gastric cancer has also been increasing. Proximal gastrectomy (PG) was recommended for the surgical treatment of early gastric cancer in the 2018 Japanese Gastric Cancer Guidelines [3]. Although the standard procedure for advanced gastric cancer of the upper stomach should be total gastrectomy, it was reported that distant side lymph node metastasis was rare if the tumor was localized to the upper stomach [4]. Therefore, proximal gastrectomy was chosen for favorable prognosis with reduced postgastrectomy symptoms and surgical invasiveness [5, 6]. However, a general consensus on the choice of surgical method for upper-third gastric cancer is lacking.

The purpose of the present study was to compare the short-term surgical outcomes between proximal gastrectomy with gastric tube-esophageal reconstruction and proximal gastrectomy with jejunal interposition reconstruction in upper-third gastric cancer.

## Materials and methods

### Patients

From June 2007 to December 2008, we retrospectively enrolled 301 patients with upper-third gastric cancer at Harbin Medical University Cancer Hospital. All cases were confirmed by pathological diagnoses through electronic gastroscopies, and we used the 7th edition of the TNM classification to evaluate the pathological staging by the Union for International Cancer Control/American Joint Committee on Cancer (AJCC) [7]. All patients underwent surgery for upper-third gastric cancer. One hundred fifty patients underwent jejunal interposition reconstruction surgery, and 151 patients underwent gastric tube reconstruction surgery. This study had the support and approval of the Medical Ethics Committee of Harbin Medical University Cancer Hospital. All procedures were performed in accordance with the standards of the 1964 Declaration of Helsinki and its later amendments.

The inclusion criteria were as follows: (1) pathologically confirmed gastric cancer; (2) tumors located in the upper third of the stomach; (3) survival time equal to or greater than 3 months; and (4) good performance status, performance status 0–2 according to the Eastern Cooperative Oncology Group criteria (ECOG) and Karnofsky performance status (KPS) ≥ 80. The exclusion criteria were as follows: (1) patients with other cancers or gastrectomy for benign disease; (2) patients who received neoadjuvant chemotherapies, radiotherapies, targeted therapy, and other anticancer treatments; (3) patients with severe comorbidities, such as myocardial infarction, respiratory disorder, liver cirrhosis or chronic renal failure; (4) patients with atrophic gastritis or intestinal metaplasia by endoscopic findings; and (5) patients without a complete follow-up record.

### Surgical techniques

Every patient underwent proximal gastrectomy. Of all patients, 150 underwent proximal gastrectomy and jejunal interposition reconstruction; 151 underwent proximal gastrectomy and gastric tube reconstruction. The grouping method was determined based on the status of the patient, which caused different nutritional statuses between the two groups. The interposed jejunal segments were 20 cm. Proximal gastrectomy was completed with dissection of the perigastric lymph nodes and lymph nodes 7, 8a, 9, and 11p according to the Japanese classification of gastric carcinoma before reconstruction [6].

### Outcome evaluation

All clinical data were collected from the medical records. Follow-up time was measured from the date of gastrectomy to three months after surgery. The clinicopathological characteristics included age; sex; height; weight; Body Mass Index (BMI); hospital stay; operating time; blood loss; tumor size; ABO blood type; pathologic T, N and M stage; Lauren classification; differentiation; vessel invasion; nerve invasion; pathology; total lymph nodes and positive lymph nodes. We used the Gastrointestinal Symptom Rating Scale (GSRS) and Visick grade to evaluate postgastrectomy syndromes. The primary endpoints of this study were the GSRS and Visick grade in both groups three months after surgery in the clinic. The assessment of the GSRS score was evaluated by the same physician who was blinded to the group assignments. The GSRS included abdominal pain (abdominal pain, nausea and vomiting), reflux (heartburn, acid regurgitation), diarrhea (diarrhea, loose stools, increased passage of stools, urgent need for defecation), indigestion (borborygmus, abdominal distension, eructation, increased flatus), and constipation (decreased passage of stools, hard stools, feeling of incomplete evacuation).

Nutritional status was assessed based on nutritional parameters, such as total protein, albumin, globulin, prealbumin, white blood cells, neutrophils, lymphocytes, monocytes, hemoglobin, erythrocytes and platelets. Postoperative gastrointestinal symptoms included abdominal pain, nausea and vomiting, heartburn, acid regurgitation, diarrhea, loose stools, increased passage of stools, urgent need for defecation, borborygmus, abdominal distension, eructation, increased flatus, decreased passage of stools, hard stools, and feeling of incomplete evacuation.

Gastrointestinal fiberoscopy was used to evaluate the prevalence and severity of reflux esophagitis. Reflux esophagitis was classified using the Los Angeles (LA) classification system [7], which included four grades as follows: Grade A, ≥ 1 mucosal breaks ≤ 5 mm long, none of which extends between the tops of the mucosal folds; Grade B, ≥ 1 mucosal breaks > 5 mm long, none of which extends between the tops of two mucosal folds; Grade C, mucosal breaks that extend between the tops of ≥ 2 mucosal folds and involve < 75% of the esophageal circumference; Grade D, mucosal breaks that involve ≥ 75% of the esophageal circumference.

### Statistical analysis

IBM SPSS Statistics 19.0 (IBM, Corp., Armonk, NY, USA) was used for the statistical analyses. Fisher’s exact test and the Chi-square test were used for categorical variables, and Student’s t test was used for continuous variables in comparison with clinical outcomes. A *P*-value < 0.05 was considered statistically significant.

## Results

### Patient characteristics

A total of 301 patients were enrolled in this study. In total, 150 patients underwent jejunal interposition reconstruction surgery (the JI group), and 151 patients underwent gastric tube reconstruction surgery (the GT group). The age of all patients ranged from 24 to 84 years. The age of the JI group ranged from 24 to 77 years, and the age of the GT group ranged from 24 to 84 years. In total, 243 patients were males, and 58 patients were females. The clinicopathological features of all patients are shown in Table 1. No significant differences in sex, weight, BMI (body mass index), blood loss, tumor size, ABO blood type, TNM stage, Lauren classification, or vessel invasion were noted between the groups.

**Table 1:** Clinicopathological characteristics of patients who underwent JI or GT reconstruction

Parameters	Total	JI group	GT group	*P* value
Cases (n)	301	150	151	
Age (years)				0.032
< 60	136	77	59	
≥ 60	165	73	92	
Gender				0.791
Male	243	122	121	
Female	58	28	30	
Height (cm)	170 ± 7	171 ± 6	168 ± 7	< 0.001**
Weight (kg)	68 ± 11	67 ± 10	68 ± 12	0.433
BMI	23.66 ± 3.75	23.28 ± 3.95	24.04 ± 3.50	0.078
Hospital stay (day)	23 ± 3	21 ± 1	25 ± 5	< 0.001**
Operating time (min)	201 ± 54	220 ± 52	182 ± 50	< 0.001**
Blood loss (ml)	162 ± 95	169 ± 84	155 ± 104	0.200
Tumor size (cm)				0.778
< 5	179	88	91	
≥ 5	122	62	60	
ABO blood type				0.745
A	78	44	34	
B	105	51	54	
O	91	42	49	
AB	27	13	14	
Pathological TNM classification				
T stage				< 0.001**
T1	34	14	20	
T2	46	21	25	
T3	96	28	68	
T4	125	87	38	
N stage				< 0.05*
N0	141	69	72	
N1	64	26	38	
N2	57	38	19	
N3	39	17	22	
TNM stage				0.093
I	65	27	38	
II	119	56	63	
III	117	67	50	
Lauren Classification				0.144
Diffuse type	18	5	13	
Intestinal type	208	108	100	
Mixed type	75	37	38	
Differentiation				< 0.05*
Poorly differentiated	126	73	53	
Moderately differentiated	143	65	78	
Well differentiated	32	12	20	
Vessel invasion				0.867
Positive	166	82	84	
Negative	135	68	67	
Nerve invasion				0.001*
Positive	204	115	89	
Negative	97	35	62	
Pathology				< 0.001**
Adenocarcinoma	211	119	92	
Mucinous carcinoma	62	15	47	
Signet ring cell carcinoma	18	12	6	
Others	10	4	6	
Total lymph nodes				< 0.001**
< 20	168	109	59	
≥ 20	133	41	92	
Positive lymph nodes				< 0.001**
< 3	154	95	59	
≥ 3	147	55	92	

*P < 0.05; **P < 0.001

The age, height, hospital stay, operating time, pathological T and N stage, differentiation, nerve invasion, pathology, total lymph nodes and positive lymph nodes were significantly higher in the JI group than in the GT group. The operation time for the JI group (220 ± 52 min) was significantly longer than that for the GT group (182 ± 50 min), although the estimated blood loss did not differ significantly between the two groups. The hospital stay for the JI group (21 ± 1 day) was less than that for the GT group (25 ± 5 day).

### Functional outcomes

The Visick grade ranged from 1 (excellent) to 4 (poor) and focused on disease-related mental states and relevant digestive tract symptoms. The GSRS score was used to evaluate the rating scale for gastrointestinal symptoms in patients with irritable bowel syndrome and peptic ulcer disease, and the score represented the current severity of the symptoms. In general, lower Visick grades and lower GSRS scores represented better postoperative recovery. The Visick grade and GSRS score were significantly greater in the GT group than in the JI group. Reflux esophagitis, abdominal distension and constipation are the three most common symptoms of gastric cancer three months after the operation as assessed by the GSRS score (Table 2).

**Table 2:** Postoperative functional outcomes

Parameters	Total	JI group	GT group	*P* value
Cases (n)	301	150	151	
Visick grade				< 0.05*
V1	201	112	89	
V2	71	29	42	
V3	19	7	12	
V4	10	2	8	
GSRS score	26.89 ± 5.06	25.58 ± 4.56	28.48 ± 5.78	< 0.001**

*P < 0.05; **P < 0.001

### Nutritional status

The preoperative and postoperative nutritional outcomes are presented in Table 3. Total protein, albumin, globulin, prealbumin, white blood cells, neutrophils, lymphocytes, monocytes, hemoglobin, erythrocytes and platelets were collected and compared. The albumin and globulin levels of the JI group were significantly greater than those of the GT group, and the levels of total protein, prealbumin, white blood cells, neutrophils, lymphocytes, monocytes, hemoglobin, erythrocytes and platelets were similar between the two groups before surgery. The globulin and neutrophil counts of the JI group were significantly greater than those of the GT group, and the total protein, albumin, prealbumin, white blood cell, lymphocyte, monocyte, hemoglobin, erythrocyte and platelet counts were similar between the two groups after surgery.

**Table 3:** Comparison of preoperative and postoperative nutritional outcomes between groups

Parameters	Total	JI group	GT group	*P* value
Cases (n)	301	150	151	
Preoperative				
Total protein (g/l)	68.62 ± 7.92	68.23 ± 6.37	69.00 ± 9.20	0.399
Albumin (g/l)	42.44 ± 4.57	43.63 ± 4.65	41.25 ± 4.18	< 0.001**
Globulin (g/l)	26.04 ± 5.02	24.62 ± 5.38	27.44 ± 4.20	< 0.001**
Prealbumin (g/l)	254.68 ± 66.74	252.91 ± 67.49	256.44 ± 66.17	0.647
White blood cell (W)	6.62 ± 2.07	6.38 ± 2.05	6.86 ± 2.07	0.044*
Neutrophils (N)	3.89 ± 1.61	3.67 ± 1.36	4.12 ± 1.80	0.015*
Lymphocyte (L)	2.01 ± 0.69	1.98 ± 0.66	2.04 ± 0.72	0.452
Monocyte (M)	0.49 ± 0.20	0.45 ± 0.20	0.52 ± 0.20	0.003*
Hemoglobin (Hb)	133.01 ± 23.44	132.81 ± 21.71	133.22 ± 25.11	0.880
Erythrocyte (E)	4.41 ± 0.59	4.39 ± 0.55	4.44 ± 0.63	0.464
Platelet (P)	239.54 ± 75.45	250.79 ± 82.61	228.36 ± 65.98	0.010*
Postoperative				
Total protein (g/l)	54.44 ± 5.72	53.64 ± 6.13	55.23 ± 5.19	0.016
Albumin (g/l)	32.57 ± 4.08	33.11 ± 4.55	32.02 ± 3.48	0.020
Globulin (g/l)	21.79 ± 4.56	20.53 ± 5.22	23.04 ± 3.38	< 0.001**
Prealbumin (g/l)	172.06 ± 44.29	170.63 ± 44.76	173.49 ± 43.92	0.576
White blood cell (W)	11.75 ± 3.65	11.61 ± 3.87	11.90 ± 3.42	0.491
Neutrophils (N)	8.07 ± 3.82	5.87 ± 3.03	10.26 ± 3.23	< 0.001**
Lymphocyte (L)	0.95 ± 0.45	1.03 ± 0.50	0.87 ± 0.37	0.002**
Monocyte (M)	0.67 ± 0.28	0.65 ± 0.28	0.70 ± 0.28	0.122
Hemoglobin (Hb)	122.47 ± 16.42	122.32 ± 17.08	122.63 ± 15.79	0.870
Erythrocyte (E)	4.07 ± 0.51	4.07 ± 0.53	4.07 ± 0.50	0.866
Platelet (P)	203.61 ± 67.51	212.42 ± 75.00	194.80 ± 58.00	0.023*

*P < 0.05; **P < 0.001

### Endoscopic findings

In the JI group, 31 patients had reflux esophagitis. Of these cases, 20 cases were Grade A, 8 cases were Grade B, and 3 cases were Grade C. In the GT group, 48 patients had reflux esophagitis. Of these cases, 30 cases were Grade A, 13 cases were Grade B, and 5 cases were Grade C. Reflux esophagitis was significantly increased in the GT group compared to the JI group. The incidence of reflux esophagitis in the two groups is presented in Table 4.

**Table 4:** Endoscopic findings of the JI and GT group

Parameters	Total	JI group	GT group	*P* value
Cases (n)	301	150	151	
Los Angeles classification		31	48	0.028
A		20	30	
B		8	13	
C		3	5	
D		0	0	

*P < 0.05

## Discussion

Radical gastrectomy remains the mainstay of curative treatment in gastric carcinoma. Total gastrectomy has been widely regarded as a standard treatment for proximal gastric cancer to achieve a tumor-free resection margin and extended lymph node dissection [8]. However, postgastrectomy syndromes, such as malnutrition, loss of body weight, alkaline reflux esophagitis, dumping syndrome, gallstones, and diarrhea, are still serious in patients with total gastrectomy. Currently, proximal gastrectomy is a function-preserving procedure and decreases postgastrectomy syndromes, such as esophagitis [9–11]. Several postgastrectomy reconstruction methods are available, and the method chosen must prevent reflux esophagitis and ensure good dietary intake and good short-term and long-term outcome results. JI and GI are two major postgastrectomy reconstruction methods in the clinic. It is necessary to explore better methods to treat upper-third gastric cancer and improve quality of life.

The operating time for the JI group was significantly longer than that for the GT group; however, the estimated blood loss did not differ significantly between the two groups. GT reconstruction required only one anastomosis, whereas JI construction required at least three anastomoses during reconstruction.

The postoperative conditions of patients who underwent gastric tube reconstruction and jejunal interposition reconstruction have been reported in many studies [12–15]. In the GT group, the Visick grade and GSRS scores were significantly higher than those of the JI group. Patients with lower Visick grades and lower GSRS scores had better postoperative quality of life. In this study, we used the LA classification system to evaluate reflux esophagitis. Severe reflux esophagitis was observed more frequently in the GT group with the frequency of grade B or C reflux esophagitis being greater than that in the JI group. We found that the interposed jejunum functioned as a buffer for gastric acid and protected the esophagus against gastric acid reflux in patients with JI construction. Many attempts have been made to reduce the frequency of reflux esophagitis by altering the reconstruction method [16, 17]. It was reported that the incidence of patients who developed stenosis in gastric tube reconstruction and jejunal interposition reconstruction was 7.1–20% and 3.1–31.8%, respectively, and the incidence of patients who developed reflux esophagitis in gastric tube reconstruction and jejunal interposition reconstruction was 5.7–30.8% and 0–33.3%, respectively [18–21]. Some studies reported that JI reconstruction was superior to GT reconstruction; however, others reported that GT reconstruction could prevent reflux esophagitis [22]. Some studies have indicated that JI with a shorter segment is advantageous for both postoperative evaluation of the remnant stomach and prevention of postoperative reflux esophagitis [23].

It has been demonstrated that proximal gastrectomy is a physiological reconstruction method that might offer advantages in maintaining nutritional status compared with total gastrectomy [24, 25]. The postoperative nutritional status was analyzed in the two groups. The albumin and globulin levels of the JI group were significantly greater than those of the GT group before surgery, and the globulin and neutrophil levels of the JI group were significantly greater than those of the GT group after surgery. These results indicate that the high complication rate, including anastomotic stenosis and reflux, may be related to poor nutritional status in the GT group. These results lead to the conclusion that the short-term nutritional status of patients who undergo JI may be superior to that of patients who undergo GT.

Several limitations in this study should be noted. First, the study was based on retrospective data collected at one institution. Second, data on long-term outcomes, such as nutritional status or survival time, were not available. Finally, the actual motor function and activities of daily living were not evaluated.

## Conclusion

In conclusion, proximal gastrectomy is a function-preserving procedure aimed at maintaining a gastric reservoir. Compared with GT construction, JI construction exhibits clear functional advantages and may provide better quality of life for patients with upper-third gastric cancer.

## Data Availability

All data generated or analyzed during this study are included in this published article.

## Abbreviations

JI: Jejunal interposition
GT: Gastric tube
GSRS: Gastrointestinal symptom rating scale
LA: Los Angeles
PG: Proximal gastrectomy
AJCC: American joint committee on cancer
ECOG: Eastern cooperative oncology group criteria
KPS: Karnofsky performance status
BMI: Body mass index
